# Clinical Presentation, Management and Outcome of adult congenital heart disease at tertiary care hospital in Pakistan

**DOI:** 10.12669/pjms.42.9.11435

**Published:** 2026-09

**Authors:** Aliya Kemal Ahsan, Usman Tauseef, Muhammad Ayyaz, Abdul Sattar Shaikh

**Affiliations:** 1 Aliya Kemal Ahsan, FCPS, Department of Pediatric Cardiology, National Institute of Cardiovascular Diseases (NICVD), Karachi, Pakistan; 2 Usman Tauseef, FCPS, Department of Pediatric Cardiology, National Institute of Cardiovascular Diseases (NICVD), Karachi, Pakistan; 3 Muhammad Ayyaz, FCPS, Department of Pediatric Cardiology, National Institute of Cardiovascular Diseases (NICVD), Karachi, Pakistan; 4 Abdul Sattar Shaikh, FCPS, Department of Pediatric Cardiology, National Institute of Cardiovascular Diseases (NICVD), Karachi, Pakistan

**Keywords:** Atrial septal defect, Cardiac catheterization, Congenital heart disease, Tetralogy of fallot, Ventricular septal defect

## Abstract

**Objective:**

To evaluate the clinical presentation, management and outcomes of adult congenital heart disease (ACHD).

**Methodology:**

This cross-sectional study was conducted at the department of pediatric cardiology and Intensive Care Unit (ICU) of NICVD, Karachi, Pakistan, from April 2021 to December 2023. Patients of either gender, aged 18 years and above and diagnosed with CHDs were analyzed. Outcomes were noted in the form of intra-operative or post-surgery mortality, and post-surgical complications.

**Results:**

In a total of 129 patients, 68 (52.7%) were male. The median age was 22.00 years. There were 10 (7.8%) ACHD cases who were newly diagnosed. The most common CHD types were found to be tetralogy of Fallot (TOF), atrial septal defect (ASD), and ventricular septal defect (VSD), noted in 32 (24.8%), 25 (19.4%), and 22 (17.1%) patients, respectively. Shortness of breath, cyanosis, and palpitation, were the most frequent complaints at the time of presentation, reported in 120 (93.0%), 48 (37.2%), and 44 (34.1%) patients, respectively. Cardiac catheterization was the most adopted procedure performed in 41 (31.8%) patients. Total correction, VSD closure, and ASD closure were performed in 9 (7.0%), 7 (5.4%), and 7 (5.4%) patients, respectively. No intra-operative or post-operative mortality was reported. The most frequently observed post-operative complications were left ventricular dysfunction, right ventricular dysfunction, biventricular dysfunction, and ventricular hypertrophy, noted in 39 (30.2%), 23 (17.9%), 6 (4.7%), and 3 (2.3%) patients, respectively.

**Conclusion:**

The most prevalent ACHD types were TOF, ASD, and VSD. Most patients required surgical or catheter-based interventions with favorable short-term outcomes.

## INTRODUCTION

In the last few decades, there has been a significant shift in the prevalence and management of congenital heart disease (CHD). Historically, the frequency of CHD has been estimated to be one in hundred live births.^1^ By the year 2000, estimates showed a prevalence of CHD as 11.89 per 1000 children, 4.09 per 1000 adults, and 5.78 per 1000 in the general population. Subsequent findings in 2010 indicated a prevalence of 6.1 per 1000 in adults.^2^

More than two-thirds of adult congenital heart disease (ACHDs) exhibit moderate to high complexity defects.^3^,^4^ Advancements in pediatric cardiovascular surgery and innovative hybrid treatment methods for CHDs have notably increased survival rates. Survival rates for most CHDs have significantly improved and are widely accepted at around 97%, signifying substantial progress in managing these conditions.^5^

Approximately 60% CHDs are detected and managed within the first year of life, with an additional 30% diagnosed during childhood and adolescence, extending up to 16 years of age.^6^ An estimated 10% of cases remain undetected until adulthood.^6^ Many CHD patients require subsequent cardiac surgeries following their initial repair in infancy, often to address residual lesions or complications.^7^ Improved survival resulting from advancements in cardiac surgery has significantly influenced the prevalence of CHDs in adults. This underscores the critical role of quality cardiac surgical care in shaping the landscape of CHD diagnosis and management throughout the lifespan. Understanding the impact of CHD on adolescents and adults is crucial. Delayed correction of CHDs can lead to irreparable damage by adolescence, making many conditions uncorrectable.^8^ This emphasizes the urgency in comprehending the disease burden within this age group and implementing aggressive measures to enhance outcomes.

In Pakistan, there is a significant research gap regarding the clinical and echocardiographic profiles of CHDs among adult patients, while not much is known about the outcomes of ACHD patients. The present study was thought to shed light on the present state of ACHD care at perhaps the leading tertiary cardiac care hospital of Pakistan. The findings of this study can help in improving the interventions and quality of care in ACHD. This study aimed to evaluate the clinical presentation, management and outcomes of ACHD.

## METHODOLOGY

This cross-sectional study was conducted at the department of pediatric cardiology and Intensive Care Unit (ICU) of NICVD, Karachi, Pakistan, from April 2021 to December 2023.

### Ethical approval:

It was obtained from Institutional Ethical Review Board (letter number: ERC-23/2021, dated: March 10, 2021). Informed and written consents were sought from all study participants.

### Inclusion criteria:

Patients of either gender, aged 18 years and above and diagnosed with CHDs. For this study, both previously diagnosed and newly diagnosed CHD cases (confirmed through transthoracic echocardiography) were considered.

### Exclusion criteria:

Patients with acquired heart diseases, cardiomyopathy, or congenital arrhythmias were excluded.

Diagnosis of CHD was established using 2-D and color Doppler transthoracic echocardiography examinations, which employed M-mode, 2-dimensional, color Doppler, pulse, and continuous wave doppler echocardiogram techniques. CHD was defined as “a gross structural abnormality of the heart or intrathoracic great vessels that is actually or potentially of functional significance, excluding the systemic great arteries and veins.” Data collection included information on gender, age, body mass index (BMI), electrocardiogram (ECG) findings, age at CHD diagnosis, and maternal history of CHDs. Clinical signs and symptoms, and CHD related complications were also documented. BMI was classified as “normal (BMI<23 kg/m^2^).^9^ All the patients were treated as per institutional protocols. Details about the type of surgical intervention (if any) during the study period were documented. Outcomes were noted in the form of intra-operative or post-surgery mortality, and post-surgical complications.

### Statistical analysis:

Data were analyzed using IBM-SPSS Statistics, version 26.0. Categorical data like gender, provincial and residential affiliations, marital status, echocardiographic and echocardiogram findings, signs and symptoms, maternal history of CHDs, CHDs related complications, NYHA classification, interventions, and outcomes were reported as frequency and percentages. Quantitative variables like age, and BMI were shown as median and inter-quartile range. Chi-square test or Fisher Exact Test were applied to compare categorical data considering p below 0.05 as significant.

## RESULTS

In a total of 129 patients, 68 (52.7%) were male. The median age was 22.00 years (20.00-28.50 years). The median age at the time of diagnosis was 5.00 years (0.50-19.50 years). The median BMI was 17.57 kg/m^2^ (14.95-20.24 kg/m^2^). Most of the patients, 97 (75.2%) belonged to Sindh province. The most common CHD types were found to be TOF, ASD, VSD, and PDA, noted in 32 (24.8%), 25 (19.4%), 22 (17.1%), and 10 (7.8%) patients, respectively. Right axis deviation was the most common ECG abnormality, observed in 54 (41.9%) patients. Baseline characteristics of ACHD patients are shown in Table-I.

**Table-I:** Baseline characteristics of patients (n=129).

Characteristics		Number (%)
Gender	Male	68 (52.7%)
	Female	61 (47.3%)
Age (years)	18-30	100 (77.5%)
	>30	29 (22.5%)
Province	Sindh	97 (75.2%)
	Punjab	10 (7.8%)
	Khyber Pakhtunkhwa	7 (5.4%)
	Gilgit	1 (0.8%)
	Balochistan	12 (9.3%)
Residence	Urban	69 (53.5%)
	Rural	60 (46.5%)
BMI (kg/m^2^)	<23	111 (86.0%)
	≥23	18 (14.0%)
Marital status	Unmarried	85 (65.9%)
	Married	44 (34.1%)
CHD Types	TOF	32 (24.8%)
	ASD	25 (19.4%)
	VSD	22 (17.1%)
	PDA	10 (7.8%)
	CoA	5 (3.9%)
	Pulmonary Stenosis	5 (3.9%)
	VSD and DORV	4 (3.1%)
	AVSD	2 (1.6%)
	DCRV	2 (1.6%)
	DORV	2 (1.6%)
	Ebstein Anomaly	2 (1.6%)
	Others	18 (14.0%)
ECG Findings	Normal	19 (14.7%)
	Right axis deviation	54 (41.9%)
	Right ventricular hypertrophy	22 (17.1%)
	Left atrial dilatation	18 (14.0%)
	Others	16 (12.4%)
NYHA Classification	1	14 (10.9%)
	2	87 (67.4%)
	3	26 (20.2%)
	4	2 (1.6%)
History of CHD in mothers		7 (5.4%)
Newly Diagnosed		10 (7.8%)

Shortness of breath, cyanosis, palpitation, and chest pain were the most frequent complaints at the time of presentation, reported in 120 (93.0%), 48 (37.2%), 44 (34.1%), and 39 (30.2%) patients, respectively (Fig.1).

**Fig.1:**
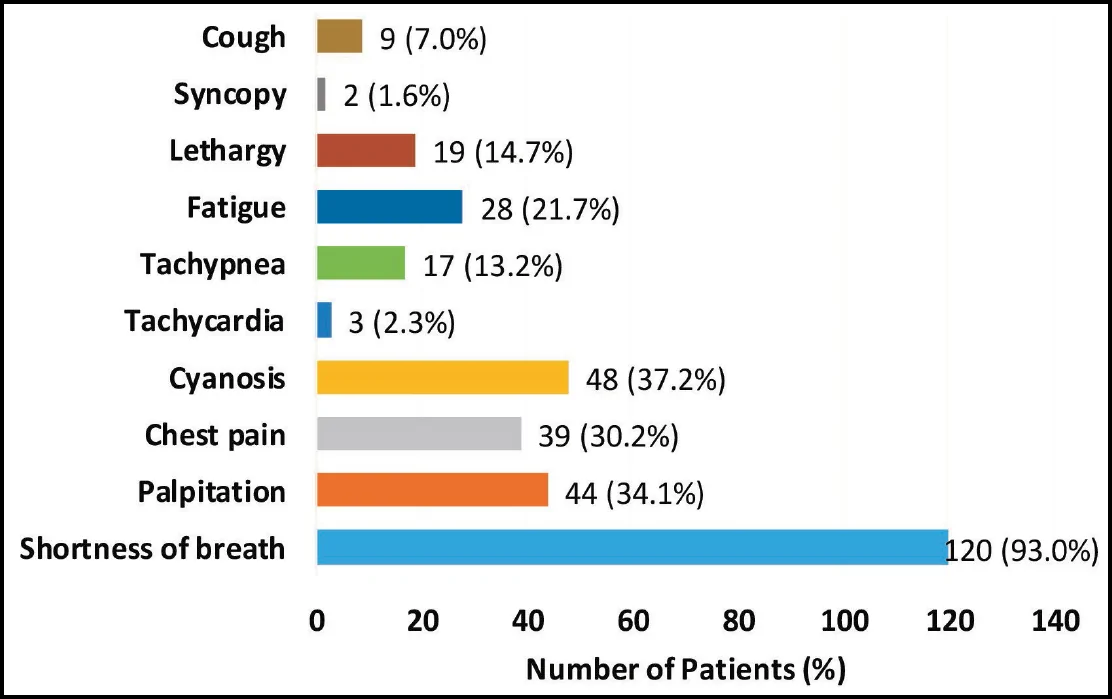
Most Frequent Signs and Symptoms (n=129).

ASD was significantly more common among females (72.0% vs. 28.0%, p=0.006). VSD had significant association with males (72.7% vs. 27.3%, p=0.039). Patients having CoA, and pulmonary stenosis were all males (p=0.031). Patients having TOF were relatively younger (p=0.041). Significantly higher proportion of patients having ASD belonged to older age groups (p<0.001). Details about the association of various CHD types with respect to gender, age and newly diagnosed cases are shown in Table-II.

**Table-II:** Association of gender, age, and newly diagnosed CHD cases with various CHD types.

CHD Types	Gender		P-value	Age (years)		P-value	Newly Diagnosed		P-value
	Male (n=68)	Female (n=61)		18-30 (n=100)	>30 (n=29)		Yes (n=10)	No (n=119)	
TOF	17 (53.1%)	15 (46.9%)	0.957	29 (90.6%)	3 (9.4%)	0.041	3 (9.4%)	29 (90.6%)	0.629
ASD	7 (28.0%)	18 (72.0%)	0.006	11 (44.0%)	14 (56.0%)	<0.001	5 (20.0%)	20 (80.0%)	0.011
VSD	16 (72.7%)	6 (27.3%)	0.039	16 (72.7%)	6 (27.3%)	0.554	-	22 (100%)	0.135
PDA	1 (10.0%)	9 (90.0%)	0.005	7 (70.0%)	3 (30.0%)	0.553	-	10 (100%)	0.340
CoA	5 (100%)	-	0.031	5 (100%)	-	0.219	1 (20.0%)	4 (80.0%)	0.296
Pulmonary Stenosis	5 (100%)	-	0.031	5 (100%)	-	0.219	-	5 (100%)	0.509
VSD and DORV	3 (75.0%)	1 (25.0%)	0.364	4 (100%)	-	0.274	-	4 (100%)	0.556
AVSD	1 (50.0%)	1 (50.0%)	0.938	2 (100%)	-	0.443	-	2 (100%)	0.679
DCRV	1 (50.0%)	1 (50.0%)	0.938	2 (100%)	-	0.443	-	2 (100%)	0.679
DORV	-	100 (100%)	0.289	2 (100%)	-	0.443	-	2 (100%)	0.679
Ebstein Anomaly	1 (50.0%)	1 (50.0%)	0.938	2 (100%)	-	0.443	-	2 (100%)	0.679

A total of 92 (71.3%) patients underwent surgical interventions. Cardiac catheterization was the most commonly adopted procedure, performed in 41 (31.8%) patients. Total correction, VSD closure, ASD closure, and PDA device closure was performed in 9 (7.0%), 7 (5.4%), 7 (5.4%), and 5 (3.9%) patients, respectively. No intra-operative or post-operative mortality was reported. Detailed distribution of various types of interventions and surgical procedures performed among ACHD patients is shown in Fig.2.

**Fig.2:**
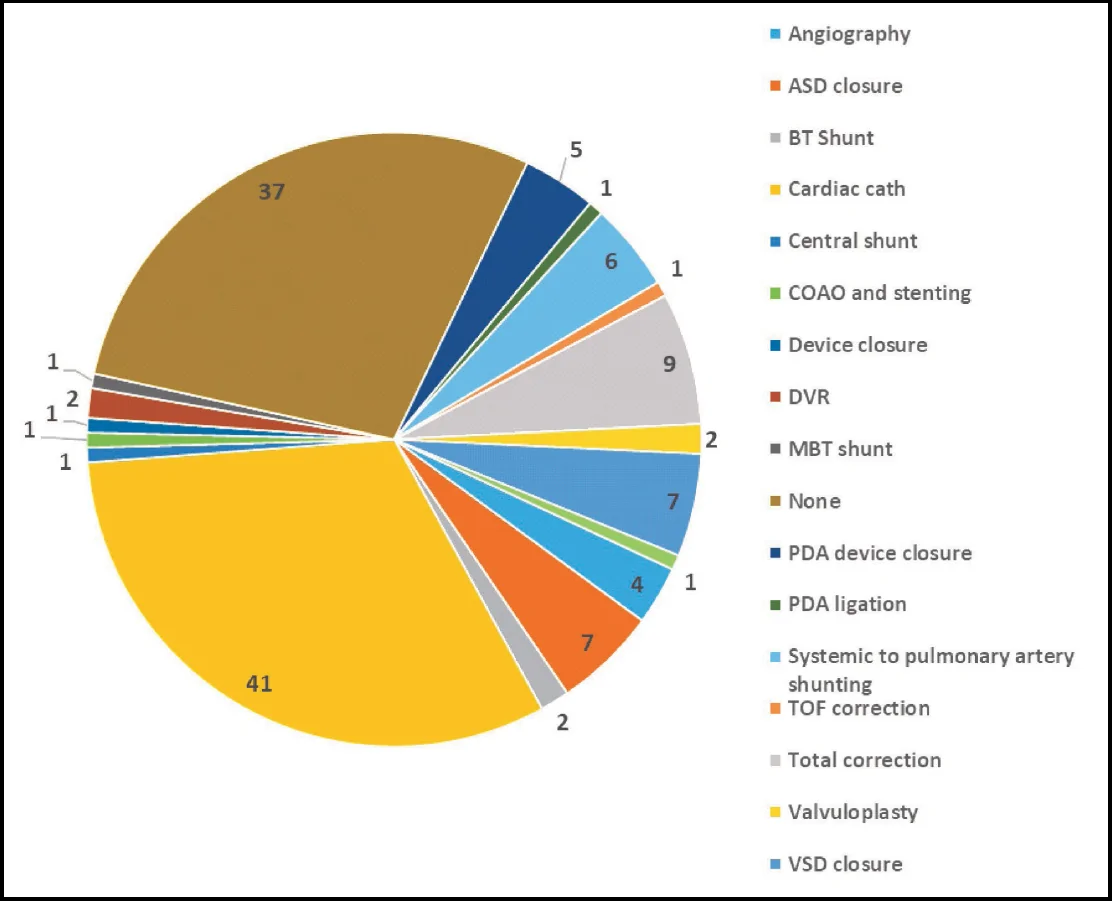
Types of Interventions / Surgical Procedures (n=129).

The most frequently observed post-surgery complications were left ventricular dysfunction, right ventricular dysfunction, biventricular dysfunction, and ventricular hypertrophy, noted in 39 (30.2%), 23 (17.9%), 6 (4.7%), and 3 (2.3%) patients, respectively (Fig.3).

**Fig.3:**
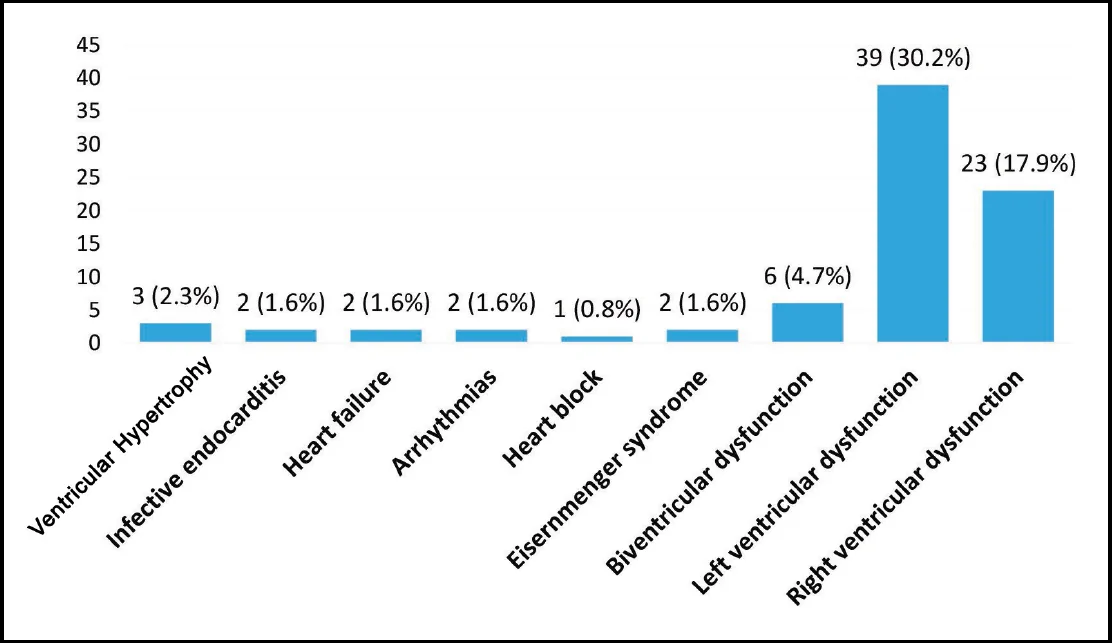
Frequency of Post-Operative Complications.

## DISCUSSION

Assessing and selecting patients with unrepaired ACHD for corrective or palliative surgery poses a significant challenge due to variation in severity and complexity of the underlying disease. In the present study, the median age at the time of presentation and at the time of diagnosis was 22.00 years (20.00-28.50 years) 5.00 years (0.50-19.50 years), respectively. A recent study from Mexico analyzing ACHD patients described the median age as 36 (28-47) years.^10^ A study from Iran reported mean age of ACHD patients to be 31.05 years.^11^ Data from Italy reported the median age of ACHD patients as 25.5 years that corresponds well with our findings.^12^

In many developing countries, congenital heart disorders often go undiagnosed during early childhood due to socioeconomic factors that hinder access to adequate prenatal care and screening.^13^ In this study, 7.8% ACHD cases were newly diagnosed. In most cases, delayed diagnosis resulted in highly complex surgeries being performed on adults with compromised cardiac health.^14^ There is a lack of timely referral to specialized centers equipped to perform these procedures once the condition is diagnosed.

Shortness of breath, cyanosis, palpitation, and chest pain were the most frequent complaints at the time of presentation, reported in 93.0%, 37.2%, 34.1%, and 30.2% patients, respectively. A community-based study conducted in Korea revealed that symptoms such as dyspnea, cyanosis, fatigue, or chest tightness were associated with CHD.^15^ Symptoms are often indicative of the clinical manifestations of CHDs and underscore the diverse and potentially serious health challenges encountered by ACHD patients.

The present study showed TOF, ASD, VSD, and PDA to be the most commonly noted CHD types, found in 24.8%, 19.4%, 17.1%, 7.8% adult patients, respectively. Globally, ASD stands out as the most prevalent type of CHD among adults.^16^ Recent local data examining children with CHDs, however, indicates that VSD and TOF are the most commonly observed CHD types.^17^ This divergence in CHD distribution between adults and pediatric populations suggests a distinct spectrum of CHD types among adults compared to children. The distribution of CHD types observed in our study differ from data reported in Canada, where researchers analyzed adult patients with CHDs. In the Canadian study, the most frequent CHD types reported were coarctation of the aorta, partial anomalous pulmonary venous return (PAPVR), TOF, and Ebstein’s anomaly.^18^ This variance in CHD distribution highlights the regional and demographic differences in the prevalence and types of CHDs among adult populations worldwide.

The literature shows that several CHD demonstrate an association with gender.^19^ Defects primarily affecting the inflow tract of the heart, such as ASD and Ebstein’s anomaly, are generally more prevalent in females.^20^,^21^ The present study showed that ASD was significantly more common among females (72.0% vs. 28.0%, p=0.006), whereas, VSD had significant association with males (72.7% vs. 27.3%, p=0.039). We found that patients having CoA, and pulmonary stenosis were all males (p=0.031). Outflow tract defects like aortic stenosis and transposition of the great arteries have been found to be more commonly observed in males.^22^ This gender discrepancy in the prevalence of specific CHD types reflects distinct embryological origins and developmental pathways. Data from the CONCOR database, which comprises information on ACHD and indicated that the gender distribution among adults with CHD mirrors the gender distribution observed at birth.^23^ This may suggest that the gender predisposition observed in specific CHD types persists into adulthood, underscoring the importance of understanding these gender-specific patterns in the management and long-term care of individuals with CHD, but this needs further verification in large scale local studies.

Interventional cardiology for ACHD has emerged as a well-established and indispensable field in its own right. It serves as a fundamental component of any healthcare center dedicated to providing comprehensive care for ACHD patients. Despite suggestions of limited safety associated with interventional procedures for the ACHD population in centers lacking global ACHD care programs, the reality is that the wide variation in clinical presentations, the emergence of novel cardiovascular pathologies with both similarities to and marked differences from both congenital and acquired conditions, and the potential for concurrent multiple organ system pathology present scenarios that are often atypical for standard adult or pediatric cardiac catheterization laboratories.^24^ These concerns, coupled with a growing recognition of the need for centralized data collection to establish robust outcomes assessments for ACHD patients undergoing interventional catheterization, underscore the importance of conducting such procedures in centers equipped with ACHD expertise and established care programs. By consolidating resources and expertise in specialized ACHD centers, healthcare providers can ensure optimal patient outcomes, minimize procedural risks, and advance our understanding of the unique challenges and complexities associated with interventional cardiology in the ACHD population.^25^

### Limitations:

This study was conducted at a single cardiovascular healthcare facility in Pakistan, which may not fully represent the diversity of the country’s population. This limits the generalizability of the findings to a broader population. The study’s sample includes only those patients who sought care at the chosen healthcare facility. Adults with CHDs who did not seek care at this facility or who may have been managed elsewhere were not included, potentially leading to selection bias. The sample size was relatively small when studying a diverse condition like CHDs. Larger sample sizes could provide more robust and generalizable results. The cross-sectional design provides a snapshot of the patient population at a specific point in time.

## CONCLUSION

The most prevalent ACHDs were TOF, ASD, VSD, and PDA. Shortness of breath, cyanosis, palpitations, and chest pain were the most frequent presenting symptoms. ASD was more prevalent in females, while VSD was more common in males. Most patients required surgical or catheter-based interventions with favorable short-term outcomes.

### Recommendations:

We only evaluated immediate outcomes among current set of patients so further studies should be planned to explore more details analyzing long term follow up data.

### Authors contribution:

**AKA:** Data collection, drafting, responsible for data’s integrity, critical revisions.

**UT:** Data collection, data analysis, critical revision.

**MA:** Literature review, drafting, data collection, critical revisions.

**ASS:** Conception and design. critical review.

All authors have read and approved the final version of manuscript for publication.
